# The kynurenine:tryptophan ratio as a predictor of incident type 2 diabetes mellitus in individuals with coronary artery disease

**DOI:** 10.1007/s00125-017-4329-9

**Published:** 2017-06-13

**Authors:** Eirik W. Rebnord, Elin Strand, Øivind Midttun, Gard F.T. Svingen, Monika H.E. Christensen, Per M. Ueland, Gunnar Mellgren, Pål R. Njølstad, Grethe S. Tell, Ottar K. Nygård, Eva R. Pedersen

**Affiliations:** 1 Department of Clinical Science, University of Bergen, Bergen, Norway; 2 KG Jebsen Centre for Diabetes Research, University of Bergen, Bergen, Norway; 3 Department of Heart Disease, Haukeland University Hospital, Jonas Lies vei 65, 5021 Bergen, Norway; 4 Bevital, Bergen, Norway; 5 Department of Medicine, Haukeland University Hospital, Bergen, Norway; 6 Laboratory of Clinical Biochemistry, Haukeland University Hospital, Bergen, Norway; 7 Hormone Laboratory, Haukeland University Hospital, Bergen, Norway; 8 Department of Pediatrics, Haukeland University Hospital, Bergen, Norway; 9 Department of Global Public Health and Primary Care, University of Bergen, Bergen, Norway

**Keywords:** Amino acid metabolism, Biomarkers, Clinical epidemiology, Type 2 diabetes

## Abstract

**Aims/hypothesis:**

The tryptophan metabolite kynurenine has potent immune modulatory and vasoactive properties. Experimental data implicate kynurenine in obesity-related morbidities. Epidemiological studies are, however, sparse. We evaluated associations of the plasma and urine kynurenine:tryptophan ratio (KTR) to incident type 2 diabetes.

**Methods:**

We followed 2519 individuals with coronary artery disease (CAD; 73.1% men) without diabetes at baseline for a median of 7.6 years, during which 173 (6.9%) new incidences of type 2 diabetes were identified. Multivariate Cox regression analyses were applied to investigate the prospective relationships of plasma and urine KTR with new onset type 2 diabetes.

**Results:**

At inclusion, mean (SD) age was 61.3 (10.4) years, BMI was 25.9 (3.71) kg/m^2^ and median (interquartile range) HbA_1c_ was 5.6% (5.0%–6.0%) (38 [31–42] mmol/mol). Plasma KTR was not significantly related to type 2 diabetes risk. By contrast, urine KTR showed a strong positive association. Comparing quartile 4 with quartile 1, the HRs (95% CIs) were 2.59 (1.56, 4.30) and 2.35 (1.39, 3.96) in the age- and sex-adjusted and multivariate models, respectively.

**Conclusions/interpretation:**

Urine KTR is a strong predictor of incident type 2 diabetes in individuals with CAD. Potential clinical implications and possible pathogenic roles of renal kynurenine excretion in type 2 diabetes development should be further elucidated.

**Electronic supplementary material:**

The online version of this article (doi:10.1007/s00125-017-4329-9) contains peer-reviewed but unedited supplementary material, which is available to authorised users.

## Introduction

Type 2 diabetes mellitus is a chronic and slowly evolving disease characterised by impaired insulin-mediated glucose uptake in peripheral tissues and a failure of the insulin secreting capacity of the pancreas [1]. While traditionally considered primarily a disorder of glucose homeostasis, it is now recognised that type 2 diabetes is associated with profound metabolic dysfunction including amino acid metabolism [1]. Further, type 2 diabetes is characterised by systemic low-grade inflammation, affecting the adipose tissue, liver and pancreas, as well as the kidneys [2].

Degradation of the essential amino acid tryptophan is closely related to immune activation. Induced by the proinflammatory cytokine IFN-γ, the enzyme indoleamine 2,3-dioxygenase (IDO1) catalyses the first and rate-limiting step of tryptophan catabolism through the kynurenine pathway [3]. Circulating levels of the metabolite kynurenine are also influenced by dietary tryptophan intake [4]. Hence, the kynurenine:tryptophan ratio (KTR) provides a more appropriate measure of tryptophan catabolism than the absolute concentration of kynurenine. In addition to reflecting the activity of the IDO1 enzyme, plasma KTR is, along with the pteridine derivative neopterin [5], a reliable indicator of IFN-γ mediated immune activation [3, 4]. Interestingly, IFN-γ has been implicated in the pathogenesis of insulin resistance [5–7] and IDO1 induction has well-characterised immunomodulatory effects [8]. Moreover, kynurenine has been identified as an endothelium-derived vasodilator [9, 10]. In experimental models, this metabolite [11, 12] and its downstream intermediates kynurenic acid [11, 13, 14] and xanthurenic acid [14] have been implicated in the pathogenesis of type 2 diabetes. Further, several plasma metabolites from the kynurenine pathway correlate with insulin resistance [15–17] and other phenotypes of the metabolic syndrome (MetS) [18]. Urinary excretion of kynurenine was increased in individuals with type 2 diabetes [19]. However, the prospective association of kynurenine pathway activation to diabetes development has not been evaluated. Therefore, we explored plasma and urine KTR as predictors of new onset type 2 diabetes in a cohort of individuals with suspected or verified stable coronary artery disease (CAD).

## Methods

### Study population

**Fig. 1:**
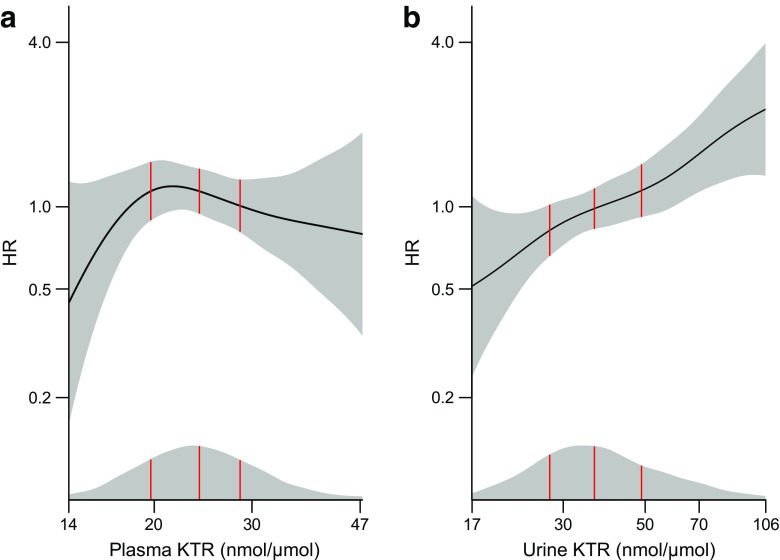
Cox regression with penalised smoothing splines showing the associations of (**a**) plasma KTR and (**b**) urine KTR to incident type 2 diabetes (*n* = 2263). The solid lines denote HRs and the grey areas, 95% CIs on the (log_e_-transformed) *y*-axes. Density plots show the distributions of (log_*e*_ transformed) plasma and urine KTR concentrations on the *x*-axes. The vertical red lines mark the 25th, 50th and 75th percentiles. Both models include adjustments for age, sex, BMI, eGFR, CRP, HbA_1c_, serum triacylglycerol, ApoA-1, urine albumin:creatinine ratio, and use of loop diuretics, ACE inhibitors or angiotensin II receptor blockers, statins and β-blockers. Ranges from the 2.5th to 97.5th percentiles of exposure variables are included

The source population has been described in detail elsewhere [20]. Briefly, it included 4164 adults, of whom 3096 were subsequently included in the Western Norway B Vitamin Intervention Trial (WENBIT) (https://ClinicalTrials.gov/ identifier: NCT00354081). The participants underwent elective coronary angiography at two Norwegian university hospitals between 2000 and 2004. For the prospective analyses, we excluded 496 individuals with a self-reported diagnosis of diabetes mellitus at baseline. We additionally omitted 42 individuals with missing HbA_1c_ records and 1107 individuals with HbA_1c_ ≥6.5% (≥48 mmol/mol), fasting glucose ≥7.0 mmol/l or non-fasting glucose ≥11.1 mmol/l. Of the 2519 eligible individuals included in the follow-up analyses, 2263 provided baseline urine samples (electronic supplementary material [ESM] Fig. 1). The study fulfilled the principles of the Declaration of Helsinki and was approved by the regional Committee for Medical and Health Research Ethics (approval number 2010/1880) and the Norwegian Data Protection Authority. All participants provided written informed consent.

### Baseline data

The procedures for collection of demographic, clinical and biochemical baseline characteristics have been described in detail elsewhere [20]. Participants were classified with CAD if coronary angiography revealed at least one significant stenosis (defined as ≥50% luminal narrowing in the main coronary arteries or major side branches). Spot urine samples were collected by the participants at home on the day of admission to hospital. Venous samples were obtained at a clinical examination before or immediately after coronary angiography. Participants reporting no intake of food or beverages during the last 6 h prior to sampling were defined as fasting. All plasma, serum and spot urine samples for the biobank were frozen at −80°C until later analysed by the Bevital laboratory (www.bevital.no). Plasma levels of kynurenine and tryptophan were measured by liquid chromatography-tandem MS, whereas the corresponding urine concentrations were analysed by GC-MS/MS. The lower limits of detection and coefficients of variability have been reported elsewhere [19, 21]. eGFR, HbA_1c_, serum lipoproteins and C-reactive protein (CRP) were calculated or measured as previously described [22]. Concentrations of individual compounds in urine were given per mol creatinine to correct for dilution. Fractional kidney excretion (FE), defined as the fraction of analyte filtered in the glomerulus that is excreted in urine, was calculated using the formula:

$$ {\mathrm{FE}}_{\mathrm{s}}=\left({\left[\mathrm{S}\right]}_{\mathrm{urine}}\times {\left[\mathrm{creatinine}\right]}_{\mathrm{plasma}}\right)/\left({\left[\mathrm{S}\right]}_{\mathrm{plasma}}\times {\left[\mathrm{creatinine}\right]}_{\mathrm{urine}}\right), $$

where S denotes kynurenine or tryptophan. HOMA2-IR and, beta cell function were calculated based on plasma glucose and serum C-peptide in a subgroup of fasting participants (*n* = 607) [23].

### Study endpoints

Information on incident type 2 diabetes was collected until 31 December 2009. We identified new diagnoses of type 2 diabetes by linkage to the Norwegian Prescription Database (NorPD, www.norpd.no). This is a national registry containing data on all dispensed drugs at outpatient pharmacies in Norway. We classified participants as having incident type 2 diabetes when receiving a first prescription of an oral glucose-lowering drug or insulin (anatomical therapeutic chemical classification system code A10). Participants were also identified with type 2 diabetes when diagnosed according to the ICD-10 (codes E11–E14; www.who.int/classifications/icd/en/) on their discharge summary following admission to a Norwegian hospital. The hospital data were obtained from the Cardiovascular Disease in Norway (CVDNOR) project (www.cvdnor.no) [24]. For the subset included in WENBIT (*n* = 1712, 68.0%), information on new onset type 2 diabetes was also obtained from self-reports and verified by glucose measurements during in-trial follow-up (2000–2005) [20].

### Statistical analyses

Variables were reported as counts (%), means (SD) or medians (interquartile range [IQR]), as appropriate. Differences in baseline characteristics according to diabetes status were evaluated using ANOVA and *χ* ^2^ analyses for continuous and categorical variables, respectively. All non-normally distributed variables were natural log (log_*e*_) transformed before being used in parametric tests.

We applied multivariate linear regression to identify covariates associated with plasma and urine KTR. In order to avoid multicollinearity not all variables from Table 1 were included in the model. The results are reported as *R* ^2^ for models and standardised β coefficients for individual variables.

**Table 1:** Baseline characteristics of the prospective study population (*n* = 2519) according to incident type 2 diabetes

Variable	Valid *n*	Total population *n* = 2519	Type 2 diabetes during follow-up		*p*
			No *n* = 2346	Yes *n* = 173	
Age (years)	2519	61.3 (10.4)	61.4 (10.4)	62.3 (10.3)	0.74
Sex (men)	2519	1841 (73.1%)	1714 (73.1%)	127 (73.4%)	0.92
Fasting	642	642 (25.5%)	605 (25.8%)	37 (21.4%)	0.30
BMI (kg/m^2^)	2519	25.9 (3.71)	25.7 (3.56)	28.1 (3.69)	<0.001
Current smoking	2519	809 (32.1%)	755 (32.2%)	54 (31.2%)	0.79
Hypertension	2519	1114 (44.2%)	1003 (42.8%)	111 (64.2%)	<0.001
Systolic BP (mmHg)	2491	139 (125–152)	139 (125–152)	140 (126–154)	0.27
Diastolic BP (mmHg)	2490	80 (75–88)	80 (75–88)	83 (76–90)	0.01
Significant CAD^a^	2519	1888 (75.0%)	1744 (74.3%)	144 (83.2%)	0.01
Renal function and inflammation					
Serum creatinine (μmol/l)	2516	89 (81–98)	89 (81–98)	91 (83–100)	0.26
eGFR (ml min^−1^ 1.73 m^−2^)	2516	91 (79–99)	91 (79–99)	91 (77–99)	0.71
Serum CRP (nmol/l)	2519	16.1 (7.90–32.4)	15.8 (7.81–31.9)	21.1 (10.3–37.0)	0.02
Plasma neopterin (nmol/l)	2504	8.09 (6.65–10.1)	8.09 (6.65–10.1)	8.11 (6.50–10.0)	0.81
Plasma kynurenine (nmol/l)	2516	1.67 (1.38–1.98)	1.66 (1.38–1.97)	1.75 (1.51–2.11)	0.001
Plasma tryptophan (μmol/l)	2516	70.3 (61.4–79.5)	70.1 (61.3–79.2)	72.5 (62.2–82.6)	0.01
Plasma KTR (nmol/μmol)	2516	23.6 (19.7–28.5)	23.5 (19.7–28.5)	23.7 (20.2–28.8)	0.18
Serum lipids					
ApoA-1 (g/l)	2519	1.31 (1.14–1.48)	1.31 (1.14–1.49)	1.24 (1.11–1.44)	0.01
ApoB (g/l)	2519	0.87 (0.73–1.05)	0.87 (0.73–1.04)	0.91 (0.76–1.08)	0.07
Triacylglycerol (mmol/l)	2516	1.44 (1.06–2.03)	1.41 (1.04–2.00)	1.70 (1.25–2.56)	<0.001
Glucose homeostasis					
Plasma glucose (mmol/l)	2518	5.4 (5.0–6.1)	5.4 (5.0–6.0)	6.3 (5.7–7.8)	<0.001
HbA_1c_ (%)	2519	5.6 (5.0–6.0)	5.6 (5.0–6.0)	5.7 (5.1–6.1)	0.16
HbA_1c_ (mmol/mol)	2519	38 (31–42)	38 (31–42)	39 (32–43)	–
Serum insulin (pmol/l)	607	21.8 (19.7–55.0)	19.7 (19.7–55.0)	39.4 (19.7–110)	0.002
Serum C-peptide (nmol/l)	607	0.71 (0.53–0.98)	0.71 (0.51–0.96)	0.91 (0.67–1.17)	0.001
HOMA2 C-peptide					
Beta cell activity	607	113 (93–138)	112 (92–138)	121 (95–149)	0.57
Insulin resistance	607	1.6 (1.2–2.2)	1.6 (1.1–2.2)	2.0 (1.5–2.7)	<0.001
Urine biomarkers					
Creatinine (mmol/l)	2263	11.7 (7.8–16.6)	11.8 (7.8–16.6)	11.3 (8.3–16.4)	0.77
Albumin:creatinine (mg/mmol)	2111	0.51 (0.37–0.82)	0.50 (0.37–0.81)	0.60 (0.42–0.99)	0.02
Kynurenine:creatinine (nmol/mmol)	2263	182 (118–277)	179 (117–275)	221 (151–322)	0.001
Tryptophan:creatinine (μmol/mmol)	2263	4.86 (3.68–6.47)	4.82 (3.68–6.46)	5.32 (3.70–6.75)	0.43
FE of kynurenine^b^	2260	8.25 (5.42–12.1)	8.15 (5.36–12.1)	9.52 (6.29–13.0)	0.03
FE of tryptophan^b^	2260	5.27 (3.81–7.08)	5.27 (3.81–7.07)	5.28 (3.83–7.26)	0.80
Urine KTR (nmol/μmol)	2263	36.3 (27.7–49.4)	36.2 (27.5–48.9)	39.8 (31.3–61.1)	<0.001
Medications					
Aspirin	2519	2084 (82.7%)	1937 (82.6%)	147 (85.0%)	0.42
Statins	2519	2020 (80.2%)	1877 (80.0%)	143 (82.7%)	0.40
β-blockers	2519	1830 (72.6%)	1696 (72.3%)	134 (77.5%)	0.14
Loop diuretics	2519	231 (9.2%)	205 (8.7%)	26 (15.0%)	0.006
Thiazides	2519	159 (6.3%)	141 (6.0%)	18 (10.4%)	0.02
ACE inhibitors and/or ARB	2519	723 (28.7%)	643 (27.4%)	80 (46.2%)	<0.001

Data are presented as *n* (%), mean (SD) or median (IQR)

^a^At least one stenosis with ≥50% luminal narrowing in a main coronary artery or its major side branches identified by coronary angiography

^b^FE = ([kynurenine]_urine_ × [creatinine]_plasma_)/([kynurenine]_plasma_ × [creatinine]_urine_)

ARB, angiotensin II receptor blockers

Statistical power was evaluated on the basis of a two-sided *t* test. At *α* = 0.05, we had a power of 100% to detect a difference of at least 15% (≥3.8 nmol/μmol) of baseline mean plasma KTR levels between participants with and without incident type 2 diabetes. For baseline urine KTR, power was 81% to detect a 15% difference (≥6.5 nmol/μmol) of mean levels.

We calculated HRs and 95% CIs for incident type 2 diabetes by Cox regression, reported per SD (log_e_ transformed) increase and for quartile (Q) 4 vs Q1 of urine and plasma KTR. The simple model was adjusted for age and sex. Additional covariates for the multivariate model were selected based on clinical relevance and included: BMI, eGFR, CRP, HbA_1c_, serum triacylglycerol, apolipoprotein (Apo) A-1, urine albumin:creatinine ratio, and the use of loop diuretics, ACE inhibitors or angiotensin II receptor blockers, statins and β-blockers. Visual inspection of survival plots did not suggest deviation from proportionality. Further, the tests for proportional hazards with Schoenfeld residuals gave *p* values of ≥0.27. Hence, we found no evidence of violation of the model assumptions.

There were 408 (16.2%) and 256 (10.2%) missing records for urine albumin:creatinine ratio and urine KTR, respectively. For all other covariates of the multivariate model the number of missing records was ≤3 (≤0.1%). Missing data were handled by listwise deletion. In secondary Cox regression, we performed multiple multivariate imputation under the assumption of missing at random. Using the fully conditional specification (iterative Markov chain Monte Carlo) method [25], 20 imputed datasets were created. All covariates of the multivariate Cox model including plasma and urine KTR, the cumulative hazard rate (Nelson–Aalen estimator) and outcome variable (dichotomous) were included in the imputation model.

**Fig. 2:**
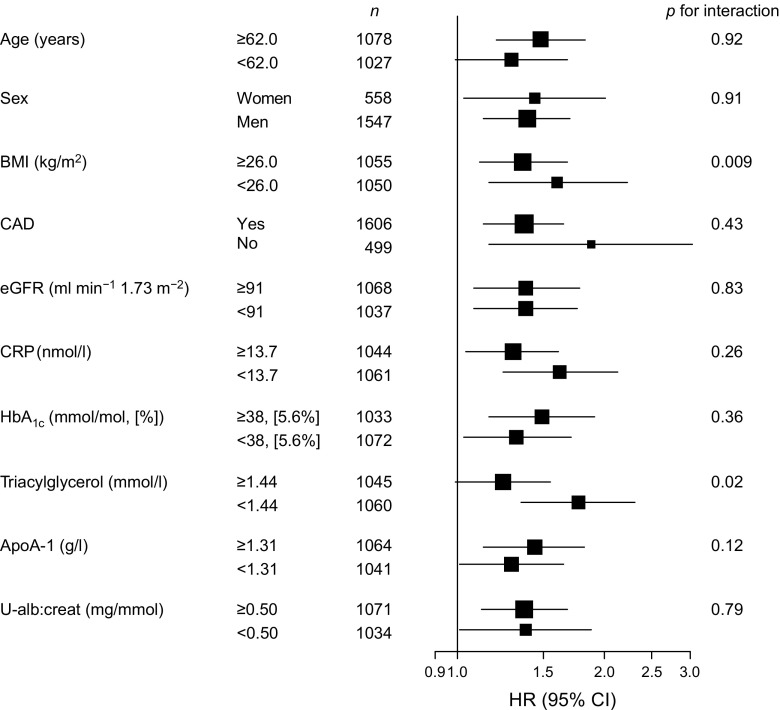
Multivariate adjusted HRs of urine KTR for incident type 2 diabetes in subgroups. Dichotomous subgroups were generated for sex and significant CAD at coronary angiography (at least one stenosis with ≥50% luminal narrowing in a main coronary artery or its major side branches, identified by coronary angiography), or according to the median values of continuous variables. HRs are represented by squares and are reported per SD increment of log_*e*_ transformed urine KTR levels. The horizontal lines indicate 95% CIs. HRs are adjusted for age, sex, BMI, eGFR, CRP, HbA_1c_, serum triacylglycerol, ApoA-1, urine albumin:creatinine (U-alb:creat) ratio, and use of loop diuretics, ACE inhibitors or angiotensin II receptor blockers, statins and β-blockers

Subgroup analyses were performed for predefined categories of categorical variables, or according to median values of continuous variables. The following covariates were included: age, sex, BMI, presence of significant CAD at coronary angiography, eGFR, CRP, HbA_1c_, serum triacylglycerol, ApoA-1 and urine albumin:creatinine. We tested effect modifications by adding interaction product terms to the models. The Benjamini–Hochberg adjustment was applied to correct for false discovery rate. However, because the subgroup analyses were planned and performed in order to facilitate the interpretation of the overall study results, *p* values for interaction are not reported adjusted for multiple comparisons in Fig. 2.

The relationships for plasma and urine KTR with incident type 2 diabetes were visualised by a 4 *df* smoothing spline fit in multivariate Cox regression models [26]. We compared model fit using Akaike’s information criterion (AIC) and explored model discrimination by calculating C statistics. By determining continuous net reclassification improvement (NRI >0) [27], we evaluated whether urine KTR improved risk classification of participants when added to the multivariate model.

All tests are two-tailed with a significance level set to 0.05. The statistical analyses were performed using SPSS Statistics version 23 (IBM, Armonk, NY, USA), SamplePower 2.0 (SPSS, Chicago, IL, USA) and R version 3.3.0 for Windows (https://www.R-project.org), and the packages ‘forest’, ‘survival’, ‘Hmisc’, ‘ROCR’ and ‘mice’.

## Results

### Baseline characteristics

From the source population (*n* = 4164), 496 (11.9%) reported a diagnosis of diabetes mellitus at inclusion; of whom the vast majority (92.5%) had type 2 diabetes. In total, 1107 (26.6%) had single measurements of plasma glucose and/or HbA_1c_ suggestive of undiagnosed diabetes. Both plasma and urine KTR levels were elevated among participants with self-reported diabetes at study enrolment. Other characteristics according to diabetes mellitus status at baseline are given in ESM Table 1.

Of the 2519 participants included in the prospective analyses, 1841 (73.1%) were men and 1888 (75.0%) had significant CAD at coronary angiography. The mean (SD) age was 61.3 (10.4) years and mean BMI was 25.9 (3.71) kg/m^2^. A total of 642 (25.5%) participants reported to be fasting at the time of sampling, with no differences in plasma or urine KTR levels according to fasting status (data not shown).

Compared with those without a diagnosis of type 2 diabetes during follow-up, participants who developed type 2 diabetes had higher BMI, and higher prevalences of hypertension and significant CAD at baseline. They were also more frequently receiving thiazides and loop diuretics. Median serum levels of CRP, triacylglycerol, plasma glucose and HOMA-IR were higher among those who developed type 2 diabetes, whereas ApoA-1 levels were lower (Table 1). Although numerically slightly higher among those with incident type 2 diabetes, neither the difference in baseline HbA_1c_ levels nor the difference in plasma KTR or neopterin levels were statistically significant. In contrast, those who subsequently developed type 2 diabetes had substantially higher levels of urine KTR (*p* < 0.001). They also had higher urine kynurenine:creatinine ratios and FE of kynurenine; however, the tryptophan:creatinine ratios and FE of tryptophan were similar between the groups (Table 1).

### Covariates associated with plasma and urine KTR

In bivariate analyses, KTR in plasma and urine were strongly related (β = 0.45, *p* < 0.001). Multivariate adjusted covariates of plasma and urine KTR are reported in Table 2. Both biomarkers were strongly positively associated with the IFN-γ marker neopterin (β = 0.46 and β = 0.28, respectively; both *p* < 0.001). Further, positive relationships were seen with age and CRP levels, whereas negative associations were found for eGFR and ApoA-1. BMI and statin use were weakly positively associated with plasma KTR, but showed no significant relation to urine KTR. As shown in Table 2, sex, presence of CAD, and the usage of loop diuretics, thiazides, ACE inhibitors, angiotensin II receptor blockers or β-blockers were not associated with KTR levels in urine or plasma. Furthermore, systolic or diastolic BP were not identified as independent covariates (data not shown). The total explained variances (*R* ^2^) of all variables in Table 2 were 48% and 25% for plasma and urine KTR, respectively.

**Table 2:** Covariates associated with plasma (*n* = 2516) and urine (*n* = 2263) KTR in multivariate linear regression analyses

Covariate	SD	Plasma (log_e_) KTR^a^ (nmol/μmol)		Urine (log_e_) KTR^b^ (nmol/μmol)	
		β^c^ (95% CI)	*p*	β^c^ (95% CI)	*p*
Age (years)	10.4	0.12 (0.08, 0.16)	<0.001	0.15 (0.11, 0.20)	<0.001
Sex (men)	–	−0.012 (−0.046, 0.022)	0.50	0.024 (−0.017, 0.066)	0.25
BMI (kg/m^2^)	3.71	0.066 (0.033, 0.099)	<0.001	0.023 (−0.017, 0.063)	0.26
eGFR (ml min^−1^ 1.73 m^−2^)	0.24	−0.22 (−0.26, −0.17)	<0.001	−0.125 (−0.178, −0.072)	<0.001
CAD^d^ (dichotomous)	–	0.015 (−0.022, 0.052)	0.43	0.021 (−0.024, 0.066)	0.36
Urine albumin:creatinine ratio (mg/mmol)	0.88	−0.009 (−0.042, 0.024)	0.59	0.043 (0.003, 0.083)	0.03
HbA_1c_ (%)	0.15	0.009 (−0.022, 0.041)	0.56	0.006 (−0.032, 0.044)	0.77
Triacylglycerol (mmol/l)	0.50	−0.001 (−0.034, 0.032)	0.95	−0.067 (−0.107, −0.027)	0.001
ApoA-1 (g/l)	0.20	−0.094 (−0.128, −0.059)	<0.001	−0.061 (−0.103, −0.019)	0.004
CRP (nmol/l)	1.08	0.039 (0.006, 0.072)	0.02	0.092 (0.052, 0.132)	<0.001
Neopterin (nmol/l)	0.37	0.46 (0.42, 0.50)	<0.001	0.28 (0.23, 0.33)	<0.001
Use of statins	–	0.047 (0.013, 0.082)	0.007	0.013 (−0.029, 0.055)	0.54
Use of loop diuretics	–	0.011 (−0.022, 0.044)	0.52	−0.028 (−0.068, 0.012)	0.18
Use of thiazides	–	−0.016 (−0.049, 0.018)	0.36	−0.006 (−0.046, 0.033)	0.75
Use of ACE inhibitors and/or ARB	–	0.019 (−0.016, 0.053)	0.28	0.0001 (−0.041, 0.041)	0.99
Use of β-blocker	–	−0.015 (−0.048, 0.019)	0.38	−0.032 (−0.073, 0.008)	0.12

^a^Multiple *R* ^2^: 48%

^b^Multiple *R* ^2^: 25%

^c^Standardised β coefficients

^d^At least one stenosis with ≥50% luminal narrowing in a main coronary artery or its major side branches identified by coronary angiography

ARB, angiotensin II receptor blockers

To evaluate the impact of insulin resistance on circulating and urinary levels of KTR, the linear regression analyses were repeated for a subset of participants for whom HOMA calculations were available (*n* = 607; data not shown). In a multivariate model including the same covariates as in Table 2, HOMA-IR was only weakly associated with plasma KTR (β = 0.09, *p* = 0.04) and showed no significant relationship to urine KTR (β = 0.05, *p* = 0.29).

### KTR and risk of type 2 diabetes

During a median (IQR) of 7.6 (6.6–8.7) years, a total of 173 (6.9%) new diagnoses of type 2 diabetes were identified. There was no significant association between plasma KTR and risk of type 2 diabetes (Fig. 1a). However, urine KTR was a strong predictor of type 2 diabetes (Fig. 1b). Comparing Q4 with Q1 of urine KTR, the HRs (95% CIs) were 2.59 (1.56, 4.30) and 2.35 (1.39, 3.96) in the age- and sex-adjusted and multivariate models, respectively (Table 3). Repeating the Cox analyses using multiple multivariate imputation provided nearly identical results (ESM Table 2). In the subset for whom calculations of HOMA indices were available (*n* = 607), urine KTR predicted new onset type 2 diabetes even after adjustment for each of the following: HOMA-B, HOMA-IR, insulin and C-peptide (ESM Table 3).

**Table 3:** HRs (95% CIs) for incident type 2 diabetes mellitus by KTR in plasma^a^ (*n* = 2516) and urine^b^ (*n* = 2263)

	Per SD increase		Q4 vs Q1	
	HR (95% CI)	*p*	HR (95% CI)	*p*
Plasma (log_e_) KTR				
Model 1^c^	1.14 (0.97, 1.33)	0.11	1.27 (0.80, 2.03)	0.31
Model 2^d^	0.99 (0.78, 1.22)	0.91	0.97 (0.54, 1.73)	0.92
Urine (log_e_) KTR				
Model 1^c^	1.39 (1.19, 1.62)	<0.001	2.59 (1.56, 4.30)	<0.001
Model 2^d^	1.38 (1.16, 1.64)	<0.001	2.35 (1.39, 3.96)	0.001

^a^SD: 0.31 nmol/μmol

^b^SD: 0.47 nmol/μmol

^c^Adjusted for age and sex

^d^Adjusted for age, sex, BMI, eGFR, CRP, HbA_1c_, serum triacylglycerol, ApoA-1, urine albumin:creatinine ratio, and the use of loop diuretics, ACE inhibitors or angiotensin II receptor blockers, statins and β-blockers

In the total population, we also observed significant associations between the urine kynurenine:creatinine ratio as well as the FE of kynurenine with risk of incident type 2 diabetes (ESM Table 4). In contrast, no such relationships were seen for tryptophan:creatinine ratio or the FE of tryptophan (ESM Table 5).

Calculated by AIC, the addition of urine KTR to the multivariate model significantly improved goodness of fit. Moreover, urine KTR provided an NRI of 0.21 (0.04, 0.38; *p* = 0.02), as well as a significant increase in the C statistic (*p* = 0.04) (ESM Table 6).

### Subgroup analyses

The multivariate association of urine KTR to new onset type 2 diabetes was further evaluated in strata of traditional diabetes risk indicators and other potential effect modifiers. Interestingly, urine KTR was a stronger predictor in subgroups having BMI and serum triacylglycerol levels below the median (Fig. 2); however, the interaction terms were not statistically significant when adjusted for multiple comparisons (*p*_*int*_ = 0.09 and *p*_*int*_ = 0.10 for BMI and serum triacylglycerol, respectively). Of note, urine KTR predicted incident type 2 diabetes both among those with and without angiographically verified CAD at baseline, with no significant effect modification according to CAD status (Fig. 2).

## Discussion

### Principal findings

In a large cohort of individuals with CAD, we observed that KTR in urine, but not in plasma, was a strong predictor of incident type 2 diabetes during 7 years of follow-up. The risk relationship was similar after extensive adjustment for potential confounders. Moreover, urine KTR significantly improved model discrimination and risk classification, both features being prerequisites for the potential clinical usefulness of a biomarker. Notably, urine KTR was an even stronger predictor among participants at presumably lower risk of type 2 diabetes evaluated by BMI and serum triacylglycerol levels.

### Strengths and limitations

Major strengths of our study include the large sample size, prospective design with long follow-up time, and detailed descriptions of clinical and biochemical baseline characteristics. The information on incident type 2 diabetes was collected from national health registries to which reporting is mandatory for all drug prescriptions and hospital admissions in Norway. There may have been some under-reporting of new onset type 2 diabetes if individuals did not receive pharmacological intervention or were not admitted to hospital during follow-up. Further, our study design did not permit the identification of individuals who actually had incident autoimmune diabetes. However, prior findings in northern European populations suggest that only 4% of individuals with newly diagnosed phenotypic type 2 diabetes may be GAD antibody positive [28]. Therefore, any misclassification is unlikely to have affected our results.

Unlike most other spot urine biomarkers, KTR does not require adjustment for urine creatinine to account for dilution. This limits the possibility of confounding, since the urinary creatinine excretion rate may be influenced by common comorbidities [29]. We have previously demonstrated good within-person reproducibility of urine KTR [19], which justifies single measurement of biomarker status [30]. Previous studies have suggested that urine tryptophan and kynurenine levels were stable in storage at room temperature for at least 48 h, representing a practical advantage in a clinical setting [31].

The study participants were all referred to hospital for elective coronary angiography. The majority had CAD, which is frequently associated with insulin resistance [32]. Notably, however, urine KTR was a strong predictor even in the subgroup without angiographic evidence of CAD. Our findings nonetheless need to be confirmed in large population-based surveys. Unfortunately, a replication cohort for plasma and urine KTR could not be provided for the present study. Future work should also evaluate urinary levels of downstream metabolites of the kynurenine pathway. As in any observational study the risk of residual confounding cannot be excluded. Further, our work does not allow firm conclusions about pathophysiological mechanisms.

### Tryptophan metabolism, type 2 diabetes and related morbidities

High levels of plasma KTR have been associated with adverse cardiovascular prognoses [33], with consistent findings in individuals with CAD [33] and in elderly people [34]. Moreover, we previously identified urine KTR as a particularly strong predictor of coronary events and death [19]. In contrast, epidemiological data on the kynurenine pathway in relation to type 2 diabetes have been sparse. Obesity has been associated with elevated plasma KTR levels, which were not normalised even after profound weight loss [6]. A cross-sectional study showed that, compared with healthy individuals, plasma KTR was increased in individuals with type 2 diabetes, and levels were even higher in those with diabetic nephropathy [35]. Moreover, urinary excretions of kynurenine [36], and its downstream metabolite xanthurenic acid, were elevated among individuals with type 2 diabetes [14]. A recent smaller case–control study reported an association of plasma tryptophan levels to new onset type 2 diabetes [37]. To the best of our knowledge, however, the relationships of plasma and urine KTR levels with incident type 2 diabetes have not previously been evaluated in a large-scale prospective setting.

### Possible mechanisms

More than 95% of tryptophan metabolism occurs through the kynurenine pathway where the first and rate-limiting step is catalysed either by the hepatic enzyme tryptophan 2,3-dioxygenase (TDO) or the ubiquitous IDO1. While TDO is constitutively active, IDO1 is induced by IFN-γ or other inflammatory cytokines. TDO is suppressed in parallel with IDO1 induction. Hence, in conditions with increased IFN-γ activity, tryptophan metabolism is shifted from the liver to extrahepatic tissues [3].

IDO1 and genes for several downstream enzymes of the kynurenine pathway were upregulated in adipose tissue of obese compared with lean women [11] and were expressed both in adipocytes and macrophages [11]. IDO1 may also be induced in pancreatic beta cells [38], as well as renal glomerular [10, 39] and tubular cells [40]. The role of tryptophan metabolism through the TDO enzyme in diabetes development is not established. TDO was not found to be increased in obese individuals [41]. In contrast, a rodent model demonstrated highly upregulated TDO expression in diet-induced obesity [42]. In our cohort, plasma and urine KTR were strongly related with plasma neopterin, indicating that IFN-γ mediated IDO1 activation is a major determinant for circulating, as well as urinary, KTR levels. However, this assumption needs to be confirmed in a properly designed experiment.

In line with previous cross-sectional findings [11, 19], we observed higher KTR levels in plasma and urine among participants with suspected or established diabetes mellitus at baseline. Further, plasma and urine KTR showed similar relationships to phenotypes of the MetS. However, in prospective analyses there was no significant association between circulating KTR levels and long-term risk of type 2 diabetes, whereas urine KTR was a strong predictor. Notably, urine KTR remained strongly associated with type 2 diabetes risk even after extensive adjustment for potential confounders.

The positive association of urine KTR with incident type 2 diabetes seems primarily to reflect elevated urinary excretion of kynurenine. The FE of tryptophan showed no significant association to type 2 diabetes development. According to previous findings, the renal elimination of kynurenine depends strongly on circulating levels [43]. Kynurenine is freely filtered in the glomeruli and undergoes nearly 100% tubular reabsorption at very low plasma concentrations but is increasingly excreted at elevated plasma levels [43]. This may suggest that the clearance of kynurenine is tightly regulated in order to balance circulating concentrations. Hence, urine KTR may potentially represent a more sensitive indicator of systemic tryptophan degradation than the corresponding plasma biomarker, but it may also reflect renal catabolism of tryptophan. Interestingly, glomerular IDO1 expression was upregulated in a rodent model of type 2 diabetes [39].

Kynurenine is an endogenous ligand of the transcription factor aryl hydrocarbon receptor [13], which mediates proinflammatory and procoagulant effects [44]. IDO1 activation has cytotoxic effects on Th1 lymphocytes [8]. Further, a recent experimental model showed that acute exposure of pancreatic islets to kynurenine augmented glucose-stimulated insulin secretion [12]. Moreover, tryptophan depletion in the microenvironment has been shown to activate the general control nonderepressible-2 (GCN2) kinase [45], which protects human glomerular endothelial cells from excessive glucose influx [46].

The kidneys are centrally involved in the pathogenesis of endothelial dysfunction [47]. Impaired nitric oxide (NO) mediated arterial vasodilation is a hallmark of the MetS and type 2 diabetes [48] as well as of cardiovascular disease (CVD) [48]. Interestingly, kynurenine has been identified as a potent endothelium-derived vasodilator, acting independently of NO [10]. Hence, elevated renal kynurenine excretion may adversely affect renal and systemic vascular function. This may partly explain the strong prognostic information from urine KTR in relation to incident type 2 diabetes in the current study, as well as to CVD, as previously shown [19].

### Clinical implications

We revealed a strong and log_e_-linear association of urine KTR with incident type 2 diabetes several years before the development of clinical disease. Prevalence data suggest that type 2 diabetes is undetected in at least 30% of cases [49]. Moreover, the development of micro- and macrovascular complications may precede the progression to overt type 2 diabetes [49]. However, several of the established risk factors for type 2 diabetes are only weakly associated with vascular events [50]. Our findings that urine KTR predicted both CVD prognosis [19] and type 2 diabetes, as well as the strong risk association among individuals not being identified by classical risk factors, encourage its further evaluation with regards to clinical application.

### Conclusions

In a large prospective cohort study of individuals with suspected or verified CAD, urine KTR was a strong predictor of incident type 2 diabetes. The roles of the tryptophan degradation pathway and renal kynurenine excretion in type 2 diabetes development should be further elucidated.

## Electronic supplementary material

ESM (PDF 1878 kb)

## Abbreviations

AIC: Akaike’s information criterion
Apo: Apolipoprotein
CAD: Coronary artery disease
CRP: C-reactive protein
CVD: Cardiovascular disease
FE: Fractional kidney excretion
IDO1: Indoleamine 2,3-dioxygenase
IQR: Interquartile range
KTR: Kynurenine:tryptophan ratio
MetS: Metabolic syndrome
NO: Nitric oxide
NRI: Net reclassification improvement
Q: Quartile
TDO: Tryptophan 2,3-dioxygenase
WENBIT: Western Norway B Vitamin Intervention Trial
