# Supplementary material for: The kynurenine:tryptophan ratio as a predictor of incident type 2 diabetes mellitus in individuals with coronary artery disease
Source: Diabetologia. 2017 Jun 13;60(9):1712–21. doi: 10.1007/s00125-017-4329-9 (PMC5552838; doi:10.1007/s00125-017-4329-9)
Supplement: Supplementary file 1 — (PDF 1878 kb) [file 125_2017_4329_MOESM1_ESM.pdf]

ESM Table 1 Baseline characteristics of the total study population (n=4164) according to diabetes mellitus at study enrolment

Variable	Valid n	Total population	Diabetes mellitus				
			No	Possible[a]	By self report		
		n=4164	n=2561	n=1107	n=496	p^b	p^c
Age (years)	4164	61.7 (10.4)	61.5 (10.4)	61.9 (10.2)	63.8 (10.2)	0.37	<0.001
Sex (men)	4164	2996 (72.0%)	1870 (73.0%)	765 (69.1%)	361 (72.8%)	0.02	0.91
Fasting	1137	1137 (27.3%)	657 (25.7%)	346 (31.3%)	134 (27.0%)	<0.001	0.53
BMI (kg/m^2)	4161	26.8 (3.98)	26.3 (3.65)	27.0 (4.09)	28.5 (4.83)	<0.001	<0.001
Current smoking	4164	1320 (31.7%)	818 (31.9%)	369 (33.3%)	133 (26.8%)	0.41	0.02
Hypertension	4155	1937 (46.5%)	1116 (43.6%)	481 (43.5%)	340 (68.5%)	0.90	<0.001
Systolic BP (mmHg)	4112	140 (126–154)	139 (125–152)	140 (127–155)	143 (130–160)	0.07	<0.001
Diastolic BP (mmHg)	4109	80 (75–88)	80 (75–88)	80 (75–89)	80 (75–88)	0.73	0.90
Significant CAD[d]	4164	3118 (74.9%)	1923 (75.1%)	774 (69.9%)	421 (84.9%)	0.001	<0.001
Renal function and inflammation							
Serum creatinine (µmol/l)	4160	89 (81–98)	89 (81–98)	88 (80–98)	90 (81–103)	0.88	0.002
eGFR (ml min^{-1} 1.73 m^{-2})	4155	91 (78–99)	91 (79–99)	90 (78–99)	90 (74–100)	0.23	0.002
Serum CRP (nmol/l)	4162	17.0 (8.29–35.0)	16.1 (8.00–32.1)	17.7 (8.48–35.9)	20.3 (10.2–46.0)	0.33	<0.001
Plasma neopterin (nmol/l)	4130	8.18 (6.67–10.4)	8.09 (6.63–10.1)	8.33 (6.82–10.7)	8.22 (6.57–11.0)	0.01	0.15
Plasma kynurenine (nmol/l)	4155	1.68 (1.39–2.01)	1.67 (1.38–1.98)	1.70 (1.39–2.03)	1.73 (1.41–2.09)	0.60	0.03
Plasma tryptophan (µmol/l)	4155	70.2 (60.7–79.7)	70.2 (61.3–79.5)	70.2 (59.5–79.9)	70.3 (60.0–80.4)	0.66	0.99
Plasma KTR (nmol/µmol)	4155	23.8 (19.8–29.0)	23.6 (19.7–28.6)	24.4 (20.1–29.4)	24.4 (19.9–30.7)	0.17	0.08

Serum lipids							
ApoA-1 (g/l)	4162	1.30 (1.13–1.48)	1.30 (1.14–1.48)	1.31 (1.13–1.49)	1.24 (1.07–1.43)	1.00	<0.001
ApoB (g/l)	4163	0.87 (0.73–1.04)	0.87 (0.73–1.05)	0.86 (0.74–1.05)	0.84 (0.72–0.98)	0.99	0.04
Triacylglycerol (mmol/l)	4160	1.50 (1.08–2.14)	1.44 (1.06–2.03)	1.51 (1.08–2.24)	1.73 (1.21–2.6)	0.004	<0.001
Glucose homeostasis							
Plasma glucose (mmol/l)	4158	5.6 (5.1–6.6)	5.4 (5.0–6.1)	5.7 (5.1–6.5)	10.0 (7.6–12.6)	<0.001	<0.001
HbA_{1c} (%)	4114	6.1 (5.4–6.9)	5.6 (5.0–6.0)	7.0 (6.7–7.5)	7.7 (6.7–9.0)	<0.001	<0.001
HbA_{1c} (mmol/mol)	4114	43 (36–52)	38 (31–42)	53 (50–58)	61 (50–75)	–	–
Serum insulin (pmol/l)	1081	28.0 (19.7–69.5)	22.8 (19.7–56.0)	23.9 (19.7–70.0)	76.8 (36.3–144)	0.33	<0.001
Serum C-peptide (nmol/l)	1081	0.77 (0.55–1.03)	0.72 (0.53–0.98)	0.80 (0.59–1.12)	0.86 (0.63–1.22)	0.002	<0.001
HOMA2 C-peptide							
Beta cell activity	1081	109 (85–137)	113 (93–138)	114 (88–141)	58 (34–87)	0.75	<0.001
Insulin resistance	1081	1.8 (1.2–2.4)	1.6 (1.2–2.2)	1.8 (1.3–2.5)	2.2 (1.7–3.4)	<0.01	<0.001
Urine biomarkers							
Creatinine (mmol/l)	3722	11.4 (7.7–16.1)	11.7 (7.8–16.5)	11.7 (7.8–16.1)	9.8 (6.9–13.8)	0.86	<0.001
Albumin:creatinine (mg/mmol)	3426	0.60 (0.40–1.10)	0.50 (0.40–0.90)	0.60 (0.40–1.10)	1.20 (0.60–3.90)	<0.001	<0.001
Kynurenine:creatinine (nmol/mmol)	3721	196 (126–305)	183 (118–278)	190 (124–299)	321 (205–486)	0.11	<0.001
Tryptophan:creatinine (µmol/mmol)	3721	5.05 (3.74–6.71)	4.86 (3.68–6.47)	5.10 (3.74–6.75)	5.90 (4.24–8.30)	0.15	<0.001
FE of kynurenine[e]	3718	8.77 (5.62–13.41)	8.27 (5.42–12.2)	8.41 (5.45–13.1)	14.0 (9.40–21.5)	0.41	<0.001
FE of tryptophan[e]	3718	5.42 (3.92–7.33)	5.28 (3.82–7.08)	5.50 (3.95–7.33)	6.22 (4.56–8.74)	0.10	<0.001
Urine KTR (nmol/µmol)	3721	37.9 (28.3–53.6)	36.4 (27.7–49.5)	36.4 (27.2–52.3)	52.1 (37.5–73.7)	0.62	<0.001

Medications							
Aspirin	4164	3400 (81.7%)	2117 (82.7%)	866 (78.2%)	417 (84.1%)	0.002	0.45
Statins	4164	3335 (80.1%)	2051 (80.1%)	860 (77.7%)	424 (85.5%)	0.10	0.01
β-blockers	4164	3018 (72.5%)	1858 (72.5%)	791 (71.5%)	369 (74.4%)	0.50	0.40
Loop-diuretics	4164	452 (10.9%)	232 (9.1%)	122 (11.0%)	98 (19.8%)	0.07	<0.001
Thiazides	4164	289 (6.9%)	160 (6.2%)	81 (7.3%)	48 (9.7%)	0.23	0.01
ACE-inhibitor and/or ARB	4164	1330 (61.5%)	733 (28.6%)	327 (29.5%)	270 (54.4%)	0.57	<0.001
Insulin	4164	142 (3.4%)	–	–	142 (28.6%)	–	–
Sulfonylurea	4164	169 (4.1%)	–	–	169 (34.1%)	–	–
Metformin	4164	193 (4.6%)	–	–	193 (38.9%)	–	–
Other glucose-lowering drugs	4164	15 (0.4%)	–	–	15 (3.0%)	–	–

Data are presented as *n* (%), mean (SD), or median (IQR)

[a]Single baseline measurement of HbA_{1c} ≥6.5% (≥48 mmol/mol) or fasting glucose ≥7.0 mmol/l or non-fasting glucose ≥11.1 mmol/l

[b]*p* No vs Possible (Games-Howell ANOVA post-hoc test and χ^2 test)

[c]*p* No vs By self report (Games-Howell ANOVA post-hoc test and χ^2 test)

[d]At least one stenosis with ≥50 % luminal narrowing in a main coronary artery or its major side branches, identified by coronary angiography

[e]$FE=([kynurenine]_{urine}\times[creatinine]_{plasma})/([kynurenine]_{plasma}\times[creatinine]_{urine})$

ARB, Angiotensin II receptor blocker

ESM Table 2 HRs (95% CIs) for incident type 2 diabetes mellitus by KTR in plasma and urine using multiple imputation[a] (n=2519)

	Per SD increase		Q4 vs Q1	
	HR (95% CI)	p	HR (95% CI)	p
Plasma ($\log_e$) KTR				
Model 1[b]	1.14 (1.11, 1.16)	0.11	1.27 (0.81, 2.01)	0.31
Model 2[c]	0.98 (0.81, 1.19)	0.86	0.85 (0.50, 1.43)	0.54
Urine ($\log_e$) KTR				
Model 1[b]	1.42 (1.22, 1.65)	<0.001	2.37 (1.40, 4.04)	0.002
Model 2[c]	1.36 (1.15, 1.60)	<0.001	2.17 (1.27, 3.71)	0.005

[a]Pooled HRs (95% CIs) from multiple imputation, using the fully conditional specification method (iterative Markov chain Monte Carlo). The imputation model includes plasma KTR, urine KTR, the outcome variable (dichotomous), the cumulative hazard rate (Nelson-Aalen estimator), and all the covariates specified in footnote [c].

[b]Adjusted for age and sex

[c]Adjusted for age, sex, BMI, eGFR, CRP, HbA_{1c}, serum triacylglycerol, ApoA-1, urine albumin:creatinine ratio, use of loop diuretics, ACE inhibitors or angiotensin II receptor blockers, statins and β-blockers

ESM Table 3 HRs (95% CIs) for incident type 2 diabetes mellitus by urine KTR[a] adjusted for parameters of glucose homeostasis (n=607)

	Per SD increase of urine (log_e) KTR	
	HR (95% CI)	p
Model[b]	1.99 (1.34, 2.96)	<0.001
Model[b] + HOMA-B	1.69 (1.08, 2.64)	0.02
Model[b] + HOMA-IR	1.52 (0.99, 2.33)	0.05
Model[b] + insulin	1.63 (1.06, 2.50)	0.03
Model[b] + C-peptide	1.53 (1.00, 2.35)	0.05

[a]SD: 0.47 nmol/µmol

[b]Adjusted for age and sex

ESM Table 4 HRs (95% CIs) for incident type 2 diabetes mellitus by urine kynurenine:creatinine[a] and FE of kynurenine[b] (n=2263)

	Per SD increase		Q4 vs Q1	
	HR (95% CI)	p	HR (95% CI)	p
Urine ($\log_e$) kynurenine:creatinine				
Model 1[c]	1.32 (1.12,1.55)	<0.001	1.95 (1.18, 3.24)	0.009
Model 2[d]	1.32 (1.11, 1.56)	0.002	1.99 (1.16, 3.44)	0.01
($\log_e$) FE of kynurenine[e]				
Model 1[c]	1.19 (1.01, 1.40)	0.04	1.57 (0.96, 2.57)	0.07
Model 2[d]	1.25 (1.05, 1.49)	0.01	1.67 (1.00, 2.79)	0.05

[a]SD: 0.63 nmol/mmol

[b]SD: 0.63

[c]Adjusted for age and sex

[d]Adjusted for age, sex, BMI, eGFR, CRP, HbA_{1c}, serum triacylglycerol, ApoA-1, urine albumin:creatinine ratio, use of loop diuretics, ACE inhibitors or angiotensin II receptor blockers, statins and β-blockers

[e]FE=$([\text{kynurenine}]_{\text{urine}} \times [\text{creatinine}]_{\text{plasma}})/([\text{kynurenine}]_{\text{plasma}} \times [\text{creatinine}]_{\text{urine}})$

ESM Table 5 HRs (95% CIs) for incident type 2 diabetes mellitus by urine tryptophan:creatinine[a] and FE of tryptophan[b] (n=2260)

	Per SD increase		Q4 vs Q1	
	HR (95% CI)	p	HR (95% CI)	p
Urine ($\log_e$) tryptophan:creatinine				
Model 1[c]	1.05 (0.89, 1.23)	0.59	1.06 (0.68, 1.68)	0.79
Model 2[d]	1.06 (0.89, 1.27)	0.49	1.07 (0.64, 1.78)	0.80
($\log_e$) FE of tryptophan[e]				
Model 1[c]	0.95 (0.80, 1.13)	0.55	1.03 (0.64, 1.63)	0.92
Model 2[d]	0.97 (0.81, 1.16)	0.74	1.00 (0.61, 1.63)	0.99

[a]SD: 0.44 nmol/mmol

[b]SD: 0.48

[c]Adjusted for age and sex

[d]Adjusted for age, sex, BMI, eGFR, CRP, HbA_{1c}, serum triacylglycerol, ApoA-1, urine albumin:creatinine ratio, use of loop diuretics, ACE inhibitors or angiotensin II receptor blockers, statins and β-blockers

[e]$FE=([tryptophan]_{urine}\times[creatinine]_{plasma})/([tryptophan]_{plasma}\times[creatinine]_{urine})$

ESM Table 6 Model fit, reclassification, and discrimination analyses for urine KTR[a] in relation to incident type 2 diabetes mellitus (*n*=2263)

	Incident type 2 diabetes					
	AIC	*p*	NRI (95% CI)	*p*	C-statistic	*p*
Model[b] without biomarker	994				0.731	
Model[b] with urine ($\log_e$) KTR	988	<0.001	0.21 (0.04, 0.38)	0.02	0.751	0.04

[a]SD: 0.47 nmol/µmol

[b]Adjusted for age, sex, BMI, eGFR, CRP, HbA_{1c}, serum triacylglycerol, ApoA-1, urine albumin:creatinine ratio, use of loop diuretics, ACE inhibitors or angiotensin II receptor blockers, statins and β-blockers

ESM Figure 1

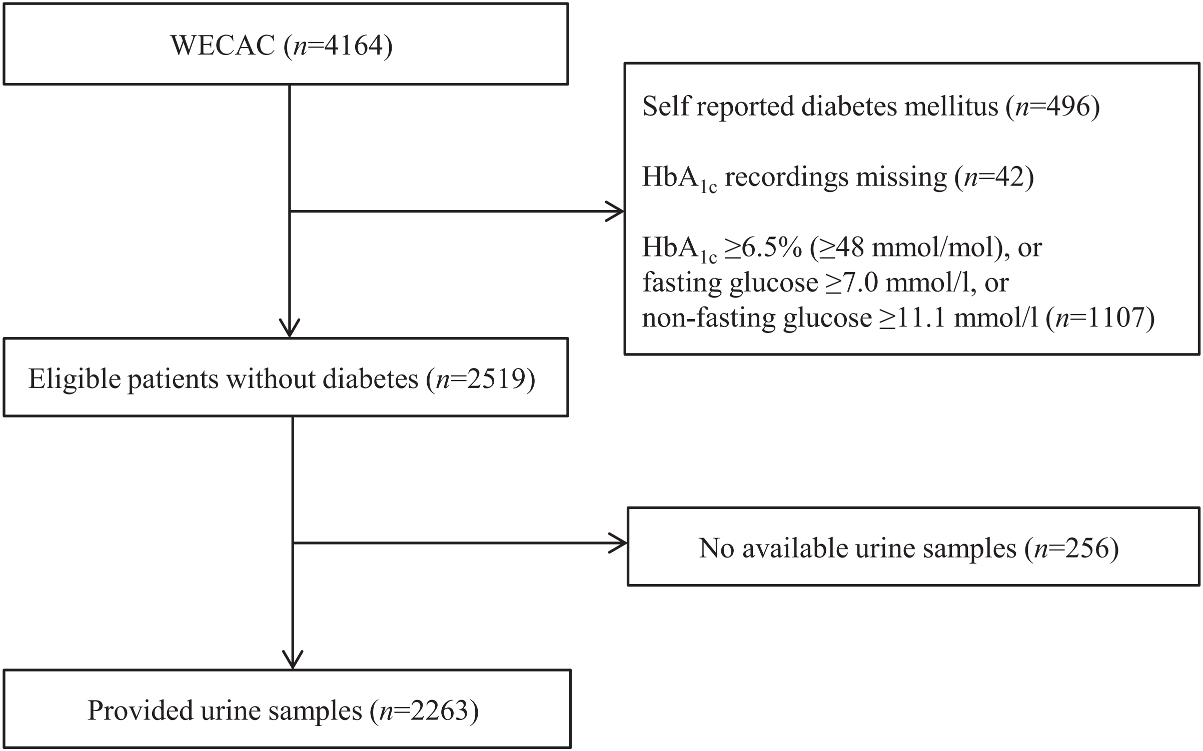

WECAC, Western Norway Coronary Angiography Cohort
